# Selenium-Chelating Peptide Derived from Wheat Gluten: *In Vitro* Functional Properties

**DOI:** 10.3390/foods13121819

**Published:** 2024-06-10

**Authors:** Yinchen Hou, Xinyang Chen, Mingyi Zhang, Shengru Yang, Aimei Liao, Long Pan, Zhen Wang, Xiaolin Shen, Xiaoqing Yuan, Jihong Huang

**Affiliations:** 1 Food and Pharmacy College, Xuchang University, Xuchang 461000, China; 80891@hnuahe.edu.cn (Y.H.); 2021930593@stu.haut.edu.cn (X.C.); 2022930656@stu.haut.edu.cn (M.Z.); aimeiliao@haut.edu.cn (A.L.); panlong@haut.edu.cn (L.P.); wangzhen@henu.edu.cn (Z.W.); 2 College of Food and Biological Engineering, Henan University of Animal Husbandry and Economy, Zhengzhou 450044, China; 81692@hnuahe.edu.cn (S.Y.); 80038@hnuahe.edu.cn (X.S.); 81685@hnuahe.edu.cn (X.Y.); 3 Collaborative Innovation Center of Functional Food by Green Manufacturing, Xuchang 461000, China

**Keywords:** selenium-chelating peptide, stability, antioxidant activity, bioavailability

## Abstract

The efficacy of selenium-chelating polypeptides derived from wheat protein hydrolysate (WPH-Se) includes enhancing antioxidant capacity, increasing bioavailability, promoting nutrient absorption, and improving overall health. This study aimed to enhance the bioavailability and functional benefits of exogenous selenium by chelating with wheat gluten protein peptides, thereby creating bioactive peptides with potentially higher antioxidant capabilities. In this study, WPH-Se was prepared with wheat peptide and selenium at a mass ratio of 2:1, under a reaction system at pH 8.0 and 80 °C. The *in vitro* antioxidant activity of WPH-Se was evaluated by determining the DPPH, OH, and ABTS radical scavenging rate and reducing capacity under different conditions, and the composition of free amino acids and bioavailability were also investigated at various digestion stages. The results showed that WPH-Se possessed significant antioxidant activities under different conditions, and DPPH, OH, and ABTS radical scavenging rates and reducing capacity remained high at different temperatures and pH values. During gastrointestinal digestion *in vitro*, both the individual digestate and the final digestate maintained high DPPH, OH, and ABTS radical scavenging rates and reducing capacity, indicating that WPH-Se was able to withstand gastrointestinal digestion and exert antioxidant effects. Post-digestion, there was a marked elevation in tryptophan, cysteine, and essential amino acids, along with the maintenance of high selenium content in the gastrointestinal tract. These findings indicate that WPH-Se, with its enhanced selenium and amino acid profile, serves as a promising ingredient for dietary selenium and antioxidant supplementation, potentially enhancing the nutritional value and functional benefits of wheat gluten peptides.

## 1. Introduction

Gluten is a natural protein extracted from wheat. It is light yellow, with a protein content of up to 80%, and is a nutritious plant protein resource. Peptide is a new type of protein hydrolysis product, derived from edible protein as a raw material, through enzymatic digestion (commercial and fermentation enzymes), followed by separation and purification [1]. In recent years, both domestic and international scholars have conducted numerous studies on the structural characterization and functional activities of peptides derived from animal, microbiological, and plant proteins [2,3]. They are responsible for signal transmission, nutrient provision, and enhancing metabolic function, playing significant roles from the cellular level to organs, systems, and overall organism behavior [4,5,6]. It has been demonstrated that peptides can be absorbed and utilized more completely than proteins. Moreover, the specific functions of peptides can vary according to the sequence and composition of amino acids. It has been observed that peptides exhibit activities such as antimicrobial, antifreeze, antioxidant, antiobesity, antidiabetic, and hypotensive properties [7,8,9].

It was the Swedish chemist Jungs Jacob Betzelius who discovered selenium in 1817 [10]. Selenium is an essential trace element involved in several important metabolic pathways in the body [11]. These pathways include thyroid hormone metabolism, the antioxidant defense system (with active centers proximate to glutathione), and immune function [12]. Selenium deficiency can lead to a number of health disorders, including Creutzfeldt–Jakob disease, cancer, heart disease, and hyperthyroidism [13,14]. Selenium has insulin-like effects in promoting cellular glucose transport, glucose metabolism, and signal transduction [15]. Selenium supplementation can improve the antioxidant capacity of brain tissue in diabetic mice, which is important for the protection of brain tissue from oxidative damage and the maintenance of normal physiological function [16]. Xu et al. [17] reported that selenium-enriched rice protein hydrolysate could dose-dependently protect against Pb^2+^-induced cytotoxicity in RAW264.7 and PC12 cells. In addition, selenium can affect the active centers of proteins and enhance their antioxidant capacity. Specifically, organic selenium can even be directly involved in scavenging free radicals [18]. Since selenium cannot be synthesized by the human body and can only be supplemented through food, developing effective food-based selenium supplementation products is highly beneficial. Selenium exists in inorganic salts which are difficult for the body to absorb [19,20]. It also has certain toxic properties, which can damage mitochondria and induce apoptosis. Inorganic selenium is frequently converted into organic selenium through chelation to ensure safe supplementation [21,22].

Bioactive peptides, characterized by potent physiological effects, high bioavailability, and diverse origins, serve as promising candidates for selenium delivery [23]. A novel product incorporating selenium and bioactive peptides synergistically provides a suitable medium for selenium supplementation, offering combined benefits of selenium and peptide activities. Currently, there is more research on chelating peptides with Fe, Zn, Ca, and other minerals, yet fewer investigations on chelating peptides of selenium, despite its considerable potential for development [24,25,26,27]. The preliminary study in our laboratory has shown that wheat protein peptides could be chelated with selenium to form chelates (WPH-Se), and it was proved that peptide–selenium chelate was formed by the N-terminal amino group, C-terminal carboxyl group, amino acid side chains, and carbon and imino groups in the peptide chain [28]. Understanding the impact of processing methods on the antioxidant activity of WPH-Se is crucial for its potential application in functional foods or nutritional health products. Gallego et al. [29] reported that the scavenging ability of Ser-Asn-Ala-Ala-Cys (SNAAC) to peroxidize free radicals and ABTS and the bleaching ability of β-carotene decreased significantly after digestion *in vitro*. Wong et al. [30] confirmed that the peptide Met-Tyr-Pro-Gly-Leu-Ala maintained excellent activity in the simulated intestinal tract. Udenigwe and Fogliano [31] suggested that many peptides easily had chemical reactions on their nucleophilic amino, carboxyl, imine, and sulfhydryl groups, which reduced the bioavailability of bioactive peptides. Therefore, further research is needed to evaluate the stability of WPH-Se under different processing conditions and gastrointestinal proteolytic enzymes to determine its potential as a selenium supplement [32]. Inorganic selenium exhibits a low absorption rate and presents poor safety of intake, whereas organic selenium possesses low toxicity and can be transported and absorbed through peptide transport channels, making it currently a hot research topic [33].

Both theoretical predictions and practical experiments are necessary to comprehensively understand the behavior of WPH-Se in the intestinal environment and its potential impact on bioavailability and efficacy. This study aimed to evaluate its antioxidant and bioavailability under various conditions, including different processing parameters such as temperature and pH. The investigation also involved analyzing its antioxidant activity, free amino acid profile, and changes in Se content during *in vitro* digestion. Therefore, this study can serve as a theoretical foundation and development efforts in utilizing WPH-Se in various functional food and nutritional formulations.

## 2. Materials and Methods

### 2.1. Materials

Porcine pancreatin (4× USP) was obtained from Sigma-Aldrich Co., Ltd. (St. Louis, MI, USA). Alkaline protease (200 U/mg) was obtained from Beijing Solarbio Science & Technology Co., Ltd. (Beijing, China) DPPH (1,1-diphenyl-2-picrylhydrazyl), ABTS (2,2′-azino-bis-3-ethylbenzothiazoline-6-sulfonic acid) diammonium salt, EDTA (ethylene diamine tetra-acetic acid), Na_2_SeO_3_, and H_2_O_2_ (*v/v*, 30%) were obtained from Shanghai Maclin Biochemical Technology Co., Ltd. (Shanghai, China) The wheat protein hydrolysate, with a degree of hydrolysis amounting to 38.72%, was prepared in the laboratory. All other chemicals were analytical grade.

### 2.2. Preparation of WPH-Se

The preparation of wheat protein hydrolysate (WPH) followed the method by Yang et al. [28]. Briefly, wheat gluten was suspended in deionized water (*m/v*, 1:9), mixed, and ultrasonicated for 20 min (55 °C, 40 kHz) (Jiangda Wukesong Biotechnology Co., Ltd., Zhenjiang, China). The mixture was extracted, adjusted to pH 8, and the enzymatic conditions were as follows: 55 °C, 2.5 h, and alkaline protease (dosage: 8000 U/g), maintained at pH 7.0~9.0 with Hcl or NaOH (1.0 M). The enzymes were subsequently inactivated by heating at 100 °C for 15 min. After cooling to room temperature, the sample was centrifuged (4000 rpm, 10 min), and the supernatant was collected and dried to WPH. WPH was suspended in a solution containing 1 mg/mL Na_2_SeO_3_ (*m/m*, 2:1). The chelation reaction between WPH and Se was conducted under specific conditions: 80 °C, pH 8.0, 120 rpm, and 60 min. Subsequently, the solution was supplemented with anhydrous ethanol at 5:1 (*v/v*) and allowed to stand for 60 min. the precipitate was dried to constant weight to obtain the WPH-Se [34].

### 2.3. Analysis of Free Amino Acids after In Vitro Digestion of WPH-Se

Free amino acids in the sample were detected following the method of Fen et al. [35] with minor modification. In brief, WPH-Se (10 mL) before and after digestion was mixed with sulfosalicylic acid (10%, *w/v*, 2.5 mL), then filtered through a 0.45 μm membrane and left at 2~8 °C for 1 h, followed by centrifugation at 4000 rpm for 15 min. The supernatant was centrifuged again at 4000 rpm for 5 min. The sample dilutions were diluted at 1:4~1:199 and filtered again through the 0.45 μm membrane for the automatic amino acid analyzer (Membra Pure A300, Hennigsdorf, Germany).

### 2.4. Effect of Temperature and pH on In Vitro Antioxidant Activity

The radical scavenging capacity of ABTS, OH (Hydroxyl), DPPH, and the reducing capacity of WPH-Se (5 mg/mL) were assessed under different temperature (25, 40, 60, 80, 100 °C) and pH levels (2, 4, 6, 8, 10).

#### 2.4.1. The DPPH Radical Scavenging Activity

The determination method of DPPH radical scavenging activity was modified according to that described by Ren et al. [36]. The scavenging activity of DPPH was calculated using the following equation.

(1) $$\mathrm{DPPH}\;\mathrm{scavenging}\;\mathrm{activity}=1\;-\;\frac{{\;A}_{\mathrm{sample}}{\;-\;A}_{\mathrm{blank}}}{A_{\mathrm{control}}}$$

A_sample_ is the absorbance value of the sample group; A_blank_ is the absorbance value of the blank sample; A_control_ is the absorbance value of the control group. All absorbances were measured at 517 nm.

#### 2.4.2. The OH Radical Scavenging Activity

The OH radical scavenging activity was evaluated by the method of Homayouni-Tabrizi et al. [37] with slight modifications. The OH radical scavenging activity of the samples was evaluated by the following equation.

(2) $$\mathrm{OH}\;\mathrm{radical}\;\mathrm{scavenging}\;\mathrm{activity}(\%)=\frac{{\;A}_{s}{\;-\;A}_{i}}{A_{0}{\;-\;A}_{i}}\times 100$$

A_s_ is the absorbance of the sample group; A_i_ is the absorbance of the control containing 1,10-phenanthroline, FeSO_4_, and H_2_O_2_; A_0_ is the absorbance of the blank containing 1,10-phenanthroline and FeSO_4_. Ultimately, the absorbance was evaluated at 536 nm.

#### 2.4.3. The ABTS Radical Scavenging Activity

The ABTS radical scavenging activity was measured based on the method of described by Yuan et al. [38] with some slight modifications. ABTS radical scavenging activity was calculated using the following equation.

(3) $$\;\mathrm{ABTS}\;\mathrm{radical}\;\mathrm{scavenging}\;\mathrm{activity}(\%)=\;1\;-\;\frac{A_{\mathrm{sample}}}{A_{\mathrm{blank}}}\times 100^{\;}$$

A_sample_ is the absorbance of the sample group; A_blank_ is the absorbance of the blank group. Ultimately, the absorbance was determined at 734 nm.

#### 2.4.4. Reducing Capacity

The reducing capacity for Fe^3+^ was measured according to the method by Siddhuraju et al. [39]. Then, the absorbance was measured at 700 nm.

### 2.5. Determination of Antioxidant Activity of WPH-Se In Vitro Digestion

The simulation utilized gastric and intestinal fluids formulated based on the TNO digestion model (Netherlands), which simulates the physiological environment of the human gastric and intestine [40,41,42]. The preparation of gastric and intestine solutions was performed using the method of Minekus et al. [43] with some modifications. Porcine gastric pepsin (0.55 g, 3000 U/mg), 0.28 g of NaCl, 0.052 g of KCl, 0.21 g of NaHCO_3_, 0.012 g of KH_2_PO_4_, 0.002 g of MgCl_2_(H_2_O)_6_, and 0.005 g of (NH_4_)_2_CO_3_ were dispersed in a 100 mL volumetric flask using deionized water; subsequently, 7.7 mL of 2 mmol/L CaCl_2_ was added. After magnetic stirring for 5 min at room temperature. Porcine pancreatin (0.25 g; 4XUSP, Sigma-Aldrich, St. Louis, MI, USA), 1 g of Pulvis fellis suis, 0.007 g of MgCl_2_ (H_2_O)_6_, 0.714 g of NaHCO_3_, 0.22 g of NaCl, 0.051 g of KCl, and 0.011 g of KH_2_PO_4_ were fixed in a 100 mL volumetric flask with deionized water, after which 7.30 mL of 9 mmol/L CaCl_2_ was added. After stirring magnetically for 10 min at room temperature, the solution was placed in the refrigerator for later use. WPH-Se solution (5 mg/mL, 20 mL) and artificial gastric fluid (20 mL) were added into the centrifuge tube, and the pH was adjusted to 2.0 with 1 M HCl, mixed well, and then oscillated in a water bath at 37 °C for 2 h. Artificial intestinal fluid (10 mL) was added, and the pH was adjusted to 6.8 with 2 M NaOH; the solution was then oscillated in a water bath at 37 °C for 0, 30, 60, 90, and 120 min, and then removed from the centrifuge tube. After that, the enzyme was inactivated by a water bath at 100 °C for 10 min and centrifuged at 4000 rpm for 10 min. Then, the ABTS, OH, and DPPH radical scavenging activities and reducing capacity were measured.

### 2.6. Statistical Analysis

Results were expressed as the mean ± standard deviation of the information obtained through triplicating calculations. Analysis of variance (ANOVA) was conducted with a *p*-value < 0.05. Multiple comparison was compared with Duncan’s test.

## 3. Results and discussion

### 3.1. Fabrication of WPH-SE

#### 3.1.1. Changes in Free Amino Acids of WPH-Se *In Vitro* Digestion

The role of bioactive peptides in the body is closely linked to their amino acid composition [44,45], which underscores the importance of studying the amino acid profile of WPH-Se at various stages of digestion, including the gastric and intestinal phases. Amino acids can be categorized based on their side chains’ chemical properties, including whether they are acidic, basic, or hydrophobic. The content of alkaline, acidic, and hydrophobic amino acids in WPH-Se accounted for 60.4%, 27.1%, and 8.5% of the total amino acid (Table 1), respectively, while essential amino acids constituted only 4.4%. Following digestion of WPH-Se in the gastric environment, an increase in alanine (Ala), cysteine (Cys), valine (Val), and leucine (Leu) was observed. Particularly, the solution exhibited a high concentration of hydrophobic amino acids. Comparative analysis of the free amino acid composition between gastric-digested and intestinal-digested WPH-Se, as well as undigested samples, revealed that the digested samples had a higher proportion of essential amino acids, especially noting the increase in tryptophan (Try), leucine (Leu), phenylalanine (Phe), and threonine (Thr) content. Zhang et al. [46] purified and identified a variety of antioxidant peptides from walnut protein hydrolysate containing specific amino acid residues such as tyrosine, cysteine, and histidine, and the amino acid composition of these antioxidant peptides plays a significant role in their antioxidant activity. Pea peptides, which contain amino acids such as tyrosine, lysine, and cysteine, have also been shown to have a strong antioxidant capacity [47]. Moreover, the nutritional value of WPH-Se was improved by a significant increase in its essential amino acid content.

#### 3.1.2. Antioxidant Activity Analysis of WHP-SE at Different Temperatures

**Figure 1:**
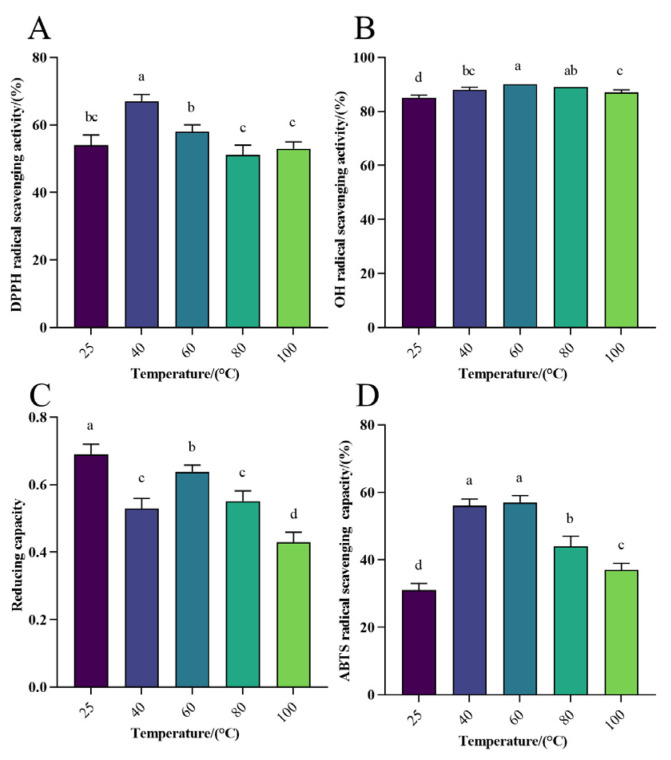
Effect of different temperatures on antioxidant activity of WPH-Se. (**A**) Effect of temperature on the scavenging rate of DPPH radicals. (**B**) Effect of temperature on OH radical scavenging rate. (**C**) Effect of temperature on the reducing capacity of Fe^3+^. (**D**) Effect of temperature on the scavenging rate of ABTS radicals. Note: Different letters indicate a significant difference (*p* < 0.05).

Thermal processing remains a common processing method widely used in the production of general foods, nutraceuticals, and pharmaceuticals, and it is essential to investigate the thermal processing characteristics of selenium-chelated peptides as a future research direction [48,49,50]. The investigation was conducted to assess the impact of varying temperatures on the antioxidant properties of WPH-Se, with 25 °C serving as the control group. The rate of DPPH radical scavenging at different temperatures was approximately 50~70% (Figure 1A). The peak scavenging rate occurred at approximately 40 °C, showing a notable increase in scavenging between 25 °C and 40 °C (*p* < 0.05). The OH scavenging rate of WPH-Se varied from 85% to 95% across different temperatures (Figure 1B). The maximum OH scavenging rate occurred at around 60 °C, evidenced by a linear increase between 25 °C and 60 °C (*p* < 0.05). Therefore, the change in temperature had little effect on the OH radical scavenging rate, highlighting the potentially efficient capacity of WPH-Se for OH radical scavenging. Furthermore, the reducing capacity of WPH-Se varied from 0.4 to 0.7 at different temperatures (Figure 1C). The highest scavenging rate was observed at approximately 25 °C, followed by a decline between 25 °C and 40 °C (*p* < 0.05), indicating that temperature variance had a significant impact on the reducing capacity of WPH-Se. Finally, the ABTS scavenging rate of WPH-Se at various temperatures ranged from 20% to 60% (Figure 1D). The scavenging rate of WPH-Se demonstrated an initial rise followed by a decline as temperature levels increased (*p* < 0.05), peaking at a rate between 40% to 60%. Thus, this indicates that temperature fluctuation has a significant effect on the ABTS scavenging rate of WPH-Se. The free radical scavenging was slightly reduced under high temperature conditions, probably due to high temperatures not causing irreversible denaturation of the peptides [51]. Instead, they might have altered the secondary structure, or prolonged exposure to high temperatures may have led to denaturation and aggregation of the antioxidant peptides, affecting their antioxidant activity and stability. This aspect warrants further exploration in future studies [52]. However, the WPH-Se prepared in this study retained a certain level of antioxidant activity at higher temperatures, indicating its potential application in thermally processed foods while maintaining good nutritional properties.

#### 3.1.3. Antioxidant Activity Analysis of WHP-Se at Different pH

**Figure 2:**
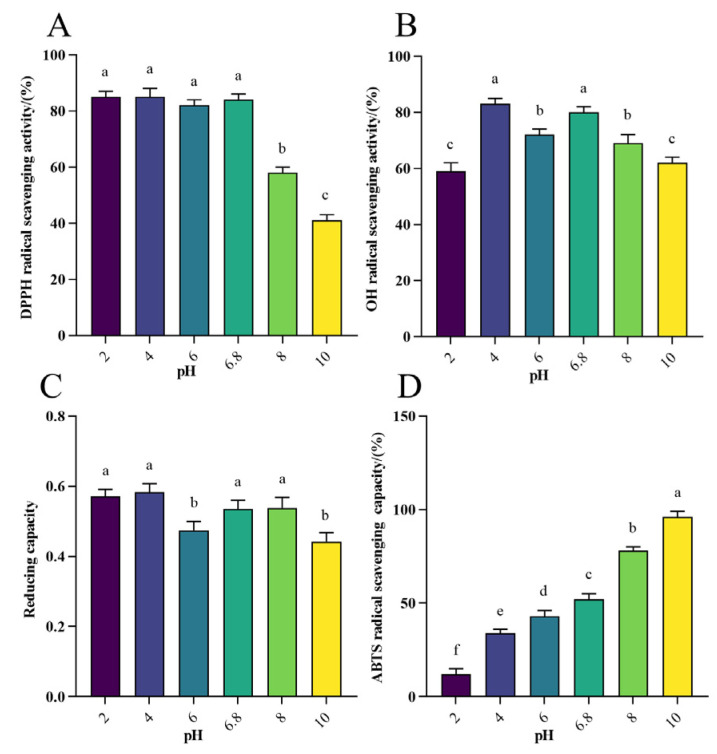
Effect of different pH on antioxidant activity of WPH-Se. (**A**) Effect of pH on the scavenging rate of DPPH radicals. (**B**) Effect of pH on OH radical scavenging rate. (**C**) Effect of pH on the reducing capacity of Fe^3+^. (**D**) Effect of pH on the scavenging rate of ABTS radicals. Note: Different letters indicate a significant difference (*p* < 0.05).

The aim of this study was to evaluate the effect of different pH on the antioxidant properties of WPH-Se, using pH 6.8 as the control. The DPPH radical scavenging rate of WPH-Se at different pH ranged from approximately 40% to 90% (Figure 2A), with the scavenging rate exceeding 80% between pH 2 and 6.8. The DPPH scavenging rate gradually decreased as the pH continued to increase (*p* < 0.05). Notably, a higher DPPH radical scavenging rate was preserved in both the gastric environment at pH 2 and the intestinal environment at pH 6.8. This observation underscores that WPH-Se exhibits noteworthy effects on the DPPH radical scavenging rate. The OH radical scavenging rate of WPH-Se ranged from approximately 50% to 80% across different pH levels (Figure 2B), with the highest rates occurring at pH 4 and pH 6.8 (*p* < 0.05). As alkalinity increased, the OH scavenging rate gradually decreased, indicating that WPH-Se possesses significant OH scavenging capacity, particularly in the intestinal environment. The reducing capacity of WPH-Se across different pH ranged from 0.4 to 0.6, with the peak observed at pH 4. This capacity remained strong at pH 2 and pH 6.8, suggesting that WPH-Se maintains effective reducing capacity in the gastrointestinal environment (Figure 2C). The ABTS radical scavenging rate of WPH-Se was varied from approximately 10% to 99% across different pH levels (Figure 2D), and the ABTS scavenging rate increased correspondingly with the increase in pH from 2 to 10 (*p* < 0.05). The ABTS radical scavenging rate was 52% at pH 6.8 and decreased to 12% at pH 2; this indicates that pH has a significant effect on the ABTS radical scavenging rate of WPH-Se. However, considering other data, WPH-Se maintains high antioxidant activity under different pH conditions. Changes in pH can alter electrostatic interactions between charged amino acids, affecting the shape of peptide molecules and leading to changes in chemical properties [53]. Acidic conditions reduce the solubility of the peptide and reduce the effectiveness of the hydroxyl group, whereas alkaline conditions reduce the DPPH radical scavenging rate of the active peptide by a wider range of factors, including racemization, deamidation, and hydrogen depletion on the hydrogen donor, and these changes may be irreversible [54,55,56]. However, ABTS radicals are mainly scavenged by antioxidant electron transfer or hydrogen protonation, and acidic groups (e.g., carboxyl groups) in peptides may be more readily protonated to their negatively charged forms under alkaline conditions [57,58]. This negatively charged state may enhance the interaction of the peptide with free radicals, thereby improving antioxidant activity.

### 3.2. Physicochemical Property Analysis of WPH-Se

#### 3.2.1. Antioxidant Activity Analysis of WPH-Se *In Vitro* Digestion

**Figure 3:**
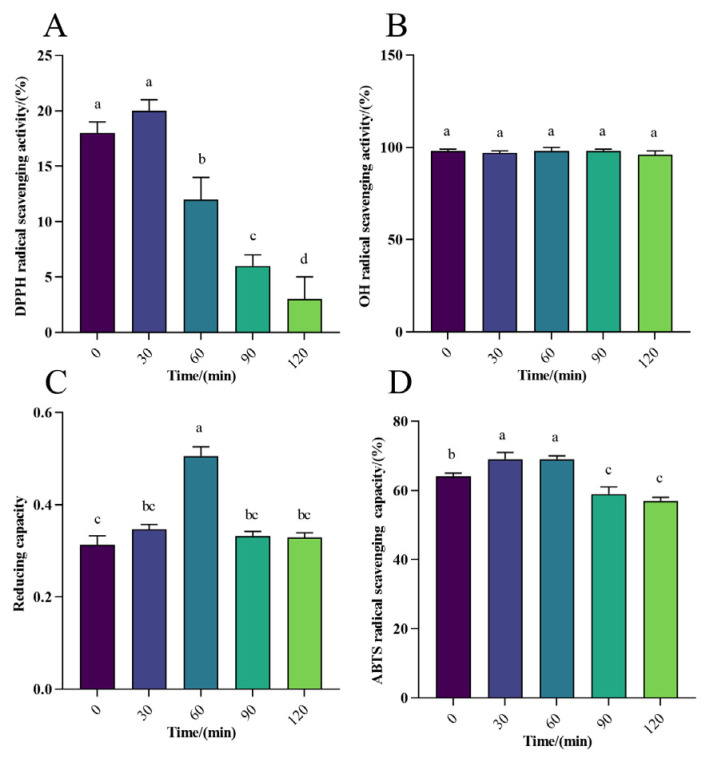
Effect of gastric digestion on antioxidant activity of WPH-SE *in vitro.* (**A**) Changes in DPPH radical scavenging activity in simulated gastric digestion of samples. (**B**) Changes in OH radical scavenging activity in simulated gastric digestion of samples. (**C**) Changes in reducing capacity of Fe^3+^ in simulated gastric digestion of samples. (**D**) Changes in ABTS radical scavenging activity in simulated gastric digestion of samples. Note: Different letters indicate a significant difference (*p* < 0.05).

**Figure 4:**
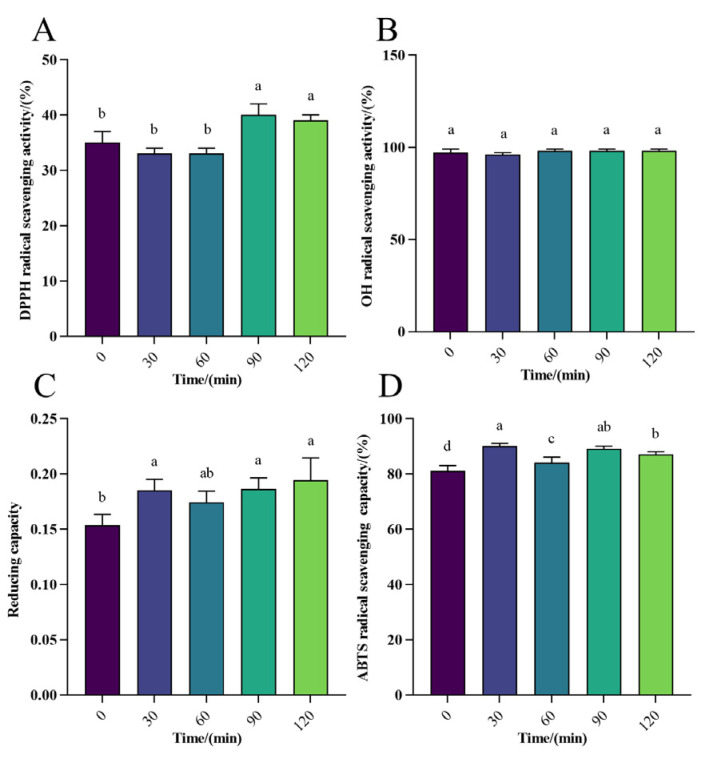
Effect of intestinal digestion on antioxidant activity of WPH-SE *in vitro.* (**A**) Changes in DPPH radical scavenging activity in simulated intestinal digestion of samples. (**B**) Changes in OH radical scavenging activity in simulated intestinal digestion of samples. (**C**) Changes in reducing capacity of Fe^3+^ in simulated intestinal digestion of samples. (**D**) Changes in ABTS radical scavenging activity in simulated intestinal digestion of samples. Note: Different letters indicate a significant difference (*p* < 0.05).

**Figure 5:**
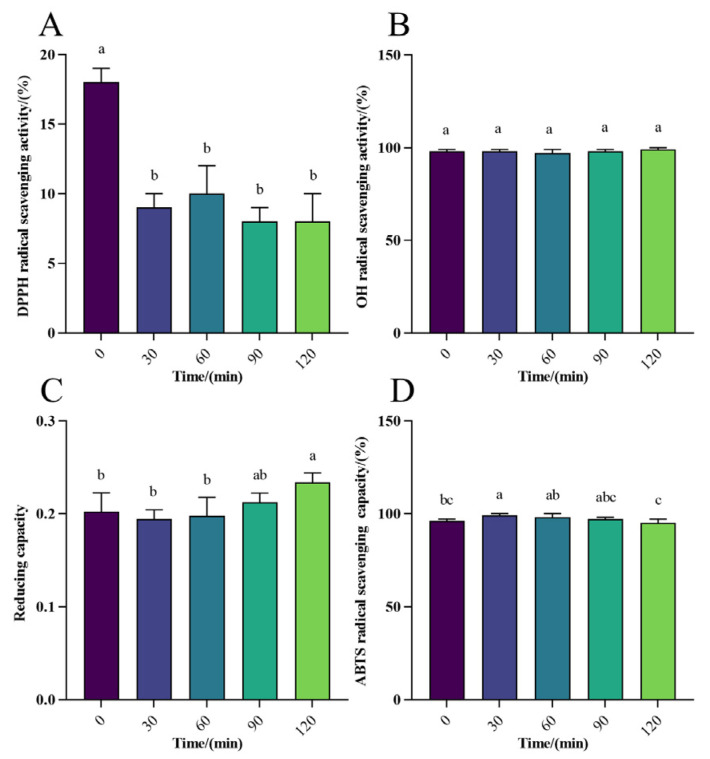
Effect of gastrointestinal digestion on the antioxidant activity of WPH-SE *in vitro.* (**A**) Changes in DPPH radical scavenging activity in simulated gastrointestinal digestion of samples. (**B**) Changes in OH radical scavenging activity in simulated gastrointestinal digestion of samples. (**C**) Changes in reducing capacity of Fe^3+^ in simulated gastrointestinal digestion of samples. (**D**) Changes in ABTS radical scavenging activity in simulated gastrointestinal digestion of samples. Note: Different letters indicate a significant difference (*p* < 0.05).

The DPPH radical scavenging rate ranged from 3% to 20% in gastric digestion *in vitro* (Figure 3A), and for intestinal digestion, it ranged from 30% to 40%, with the peak reaching 40% (Figure 4A), indicating a moderate scavenging rate of WPH-Se in the intestine compared to a lower rate in the gastric environment. However, the overall scavenging level was marginally higher than that in the gastric. With increasing digestion time (Figure 5A), the DPPH radical scavenging rate in the gastric initially decreased, reaching a maximum at 30 min. Conversely, the DPPH radical scavenging rate initially decreased and then increased over time in the intestine. Although the scavenging rate could reach 40% after 2 h, it declined with further digestion time following intestinal treatment. Overall, DPPH scavenging of WPH-Se in the intestinal tract showed a decline with prolonged digestion time. The OH scavenging of WPH-Se was approximately 100% (Figure 3B, Figure 4B, and Figure 5B), demonstrating consistent efficacy in both the gastric and intestinal digestion stages. This high performance indicates that our screened WPH-Se possesses a stable structure. Consequently, it has potential applications in treating health problems associated with hydroxyl radicals, including oxidative stress, inflammation, and atherosclerosis [59,60,61]. However, these applications will require validation through further cellular and animal experiments. The reducing capacity of WPH-Se initially increased in the gastric with time, reaching its peak at 60 min, and then decreased over time (Figure 3C). Conversely, the reducing capacity in intestinal digestion increased more slowly with time (Figure 4C); the reducing capacity of WPH-Se that underwent gastrointestinal digestion exceeded the reducing capacity of WPH-Se that was digested only in the gastric (Figure 5C). It is evident that the reducing capacity of WPH-Se is compromised after exposure to the gastric, leading to an overall reducing capacity of WPH-Se in the intestinal tract remaining at a moderate level. The ABTS radical scavenging rate was initially increased in gastric digestion and then decreased over time, reaching its peak at 60 min, and ranging from 59% to 69% (Figure 3D). Similarly, the scavenging rate was also increased initially in the intestine, reaching its maximum at 30 min, and ranging from 81% to 90% (Figure 4D). Subsequently, the ABTS radical scavenging rate of WPH-Se after gastric and intestinal digestion exceeded the ABTS radical scavenging rate of single gastric and intestinal fluids (Figure 5D), suggesting that the ABTS radical scavenging rate was significantly improved during the intestinal digestion phase, with ABTS scavenging rates ranging from 95% to 99%. The observed increase in the ABTS radical scavenging rate of WPH-Se during the digestion process is primarily attributed to enzymatic degradation by enzymes such as pepsin, trypsin, and others. Through this degradation, more antioxidant active sites are exposed, facilitating the formation of small-molecular-weight peptides and free amino acids [62]. Consequently, these compounds exhibit an enhanced ability to react with ABTS radicals, leading to a significant boost in antioxidant activity. The observed low DPPH radical scavenging rate may be attributed to pH-induced alterations in the amino acid bioactivity of the enzymatically treated WPH-Se. Interestingly, under non-enzyme conditions, it demonstrated sustained high radical scavenging rates.

With further investigation into the targeted delivery of active peptides, researchers have found that these peptides can maintain their intact structures for targeted intestinal release [63,64,65]. Therefore, we tested the antioxidant activity of WPH-Se encapsulated in carriers such as lipid particles, chitosan, and cyclodextrins, focusing specifically on its release in intestinal fluid. This encapsulation method allowed for targeted release in the intestines, where WPH-Se demonstrated effective antioxidant properties. In conclusion, it was demonstrated that WPH-Se resists gastrointestinal digestion, thereby maintaining its structural integrity. Additionally, WPH-Se exhibited notable adaptability to varying pH levels. Specifically, the ABTS radical scavenging rate, which measured the ability to neutralize free radicals, was initially lower during the gastric fluid digestion stage but increased significantly during the intestinal fluid digestion phase.

#### 3.2.2. Changes in Selenium Content before and after WPH-Se *In Vitro* Digestion

**Figure 6:**
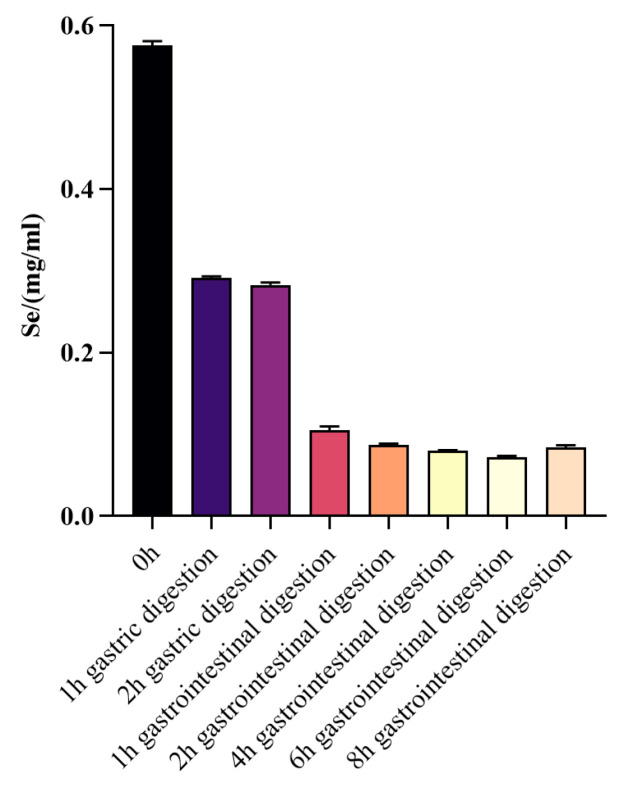
Changes in free selenium during gastrointestinal digestion *in vitro*.

The data showed that the selenium content in a solution (5 mg/mL) of WPH-Se was 0.577 mg/mL, corresponding to 11.54% of the total solution concentration (Figure 6). After gastric digestion, nearly half of the selenium in WPH-Se remains free, suggesting some degree of precipitation. The slightly acidic environment of the intestine, with pH 6.8, facilitates the dissolution and absorption of WPH-Se. Once precipitated in the gastric, WPH-Se is resolubilized in the intestine, facilitating the release and absorption of free selenium. Previous studies indicated that organic forms of selenium, typically bound to natural proteins or amino acids, are better absorbed and utilized by the human body compared to inorganic forms [66,67]. This suggests that WPH-Se may be beneficial for enhancing selenium absorption in the human body, potentially contributing to the development of effective selenium supplements.

## 4. Conclusions

This study rigorously investigated the antioxidant properties of WPH-Se under varying environmental conditions, including pH, temperature, and different stages of gastrointestinal digestion. It was found that WPH-Se retains high antioxidant activity over a broad temperature range, suggesting its potential for thermally processed foods without compromising their nutritional value. Furthermore, WPH-Se demonstrated high adaptability of its antioxidant capacity across various pH levels. Different stages of *in vitro* digestion revealed that WPH-Se maintained considerable stability during gastrointestinal digestion. Although there was a reduction in the DPPH scavenging rate, the rates for OH and ABTS scavenging remained elevated. Importantly, the maximum free radical scavenging rate of WPH-Se was higher post-intestinal fluid digestion compared to post-gastric fluid digestion, indicating not only preservation but also enhancement of its antioxidant function under intestinal conditions. This enhancement is essential for the effective antioxidant impact of WPH-Se *in vivo*. Additionally, a significant increase in the proportion of essential amino acids, particularly tryptophan (Try) and threonine (Thr), was observed during gastrointestinal digestion. The ability of WPH-Se to efficiently maintain and release bioavailable selenium after digestion further underscores its potential as a beneficial dietary supplement, enhancing selenium absorption in the human body. This comprehensive analysis confirms the versatility and effectiveness of WPH-Se as a robust antioxidant agent, suitable for various applications aimed at improving health through enhanced dietary antioxidant intake.

## Figures and Tables

**Table 1:** Changes in free amino acids after intestinal digestion of WPH-Se.

Amino Acid Name	WPH-Se (%)	Gastric WPH-Se (%)	Intestinal WPH-Se (%)
Aspartate	6.3	4.9	3.4
Threonine	1.2	2.5	2.5
Serine	2.7	5.5	3.2
Glutamate	20.8	12.5	7.0
Alanine		2.4	
Glycine	8.5	4.7	8.7
Cysteine		4.5	3.6
Valine		7.6	5.8
Leucine		4.9	
Phenylalanine			5.1
Histidine	42.3	41.4	42.5
Tryptophan			1.2
Lysine	3.2	3.1	6.7
Arginine	14.9	5.9	10.4
Acidic amino acids	27.1	17.4	10.4
Alkaline amino acids	60.4	50.4	59.6
Essential amino acids	46.7	59.5	63.8
Hydrophobic amino acids	8.5	19.6	20.8
Aromatic amino acids	0	0	6.3

## Data Availability

The original contributions presented in the study are included in the article material, further inquiries can be directed to the corresponding author.
